# Massive pulmonary embolism post sleeve gastrectomy treated with systemic thrombolytic: A case report

**DOI:** 10.1002/ccr3.8211

**Published:** 2023-11-14

**Authors:** Nader Moeinvaziri, Alireza Sadeghi

**Affiliations:** ^1^ Minimally Invasive Surgery Research Center Shiraz University of Medical Sciences Shiraz Iran

**Keywords:** alteplase, bariatric surgery, pulmonary embolism, sleeve gastrectomy, thrombolytic

## Abstract

Prompt diagnosis and management of massive pulmonary embolism after bariatric surgery is crucial, but thrombolytic therapy may increase the risk of complications such as anastomotic leakage and bleeding. Individualized management is needed.

## Graphical abstract

The medical team engaged in a lively discourse surrounding a case involving a massive pulmonary embolism that occurred within a day following sleeve gastrectomy. The central point of contention was the potential use of systemic thrombolytic treatment, which, if administered, could lead to severe complications deemed potentially fatal for a patient with recent gastrointestinal surgery.

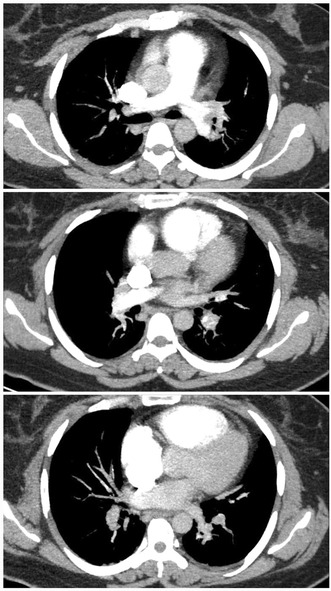

## 1 INTRODUCTION

Perioperative deep vein thrombosis (DVT) and pulmonary embolism (PE) occur in up to 5.4% and 6.4% of bariatric surgeries, respectively.^1^, ^2^, ^3^, ^4^ Although uncommon, these complications are potentially life‐threatening and are considered significant causes of mortality in bariatric surgery candidates.^5^

Due to a lack of evidence, diagnostic and therapeutic approaches in fragile postoperative settings are as crucial as uncertain. These complications are not common; thus, if they occur, physicians may be unrehearsed. Therefore, detailed discussion and sharing experiences are of great importance. Here we present a real case of acute massive PE after laparoscopic sleeve gastrectomy.

## 2 CASE PRESENTATION

A 37‐year‐old Iranian female, with a known history of hypothyroidism and class III obesity (150 cm, 95 kg, and BMI: 42.2 kg/m^2^), was admitted to our hospital for laparoscopic gastric sleeve surgery. She had a previous surgical history of three caesarian sections; the last one was almost 7 years earlier than this admission. She was on levothyroxine and metformin (self‐prescribed to lose weight) and did not use birth control. She had no family history of inheritable coagulopathies, and her social history was unremarkable.

Laboratory and clinical preoperative evaluations were unremarkable, including anesthesia, endocrinology, and cardiology. The patient was in the euthyroid state in preoperative evaluations. For thromboprophylaxis, compression stockings were applied, and she received a single dose of 5000 IU subcutaneous heparin 1 h before the surgery, according to the local guidelines. Her operation was uneventful, and sleeve gastrectomy was conducted in 100 min with six 60‐mm purple endostaplers. At the end of the surgery, a Jackson‐Pratt (JP) drain was inserted at the surgery site. She recovered, returned to the ward, and prophylactic enoxaparin (60 mg/day subcutaneously) started within 6 h after surgery. The patient was ambulated as soon as she became conscious and hemodynamically stable. The first postoperative night was uneventful, but she fainted the next morning after ambulation. She was tachycardic (pulse rate 140 bpm) and hypotensive (systolic blood pressure 80 mmHg). JP drain did not contain the bloody discharge. After primary resuscitations, considering myocardial infarction, anastomotic leakage, and PE as top differential diagnoses, the following evaluations were initiated:

- Abdominopelvic sonography showed minimal free fluid in the abdominal cavity.
- Echocardiography showed a dilated right ventricle and atrium with 60 msec pulmonary acceleration time.
- Spiral computed tomography (CT) scan of the abdomen and pelvis with intravenous contrast showed (1) a slightly edematous liver, which could be due to right‐side heart failure; and (2) mild free fluid in the abdominopelvic cavity.

**FIGURE 1:**
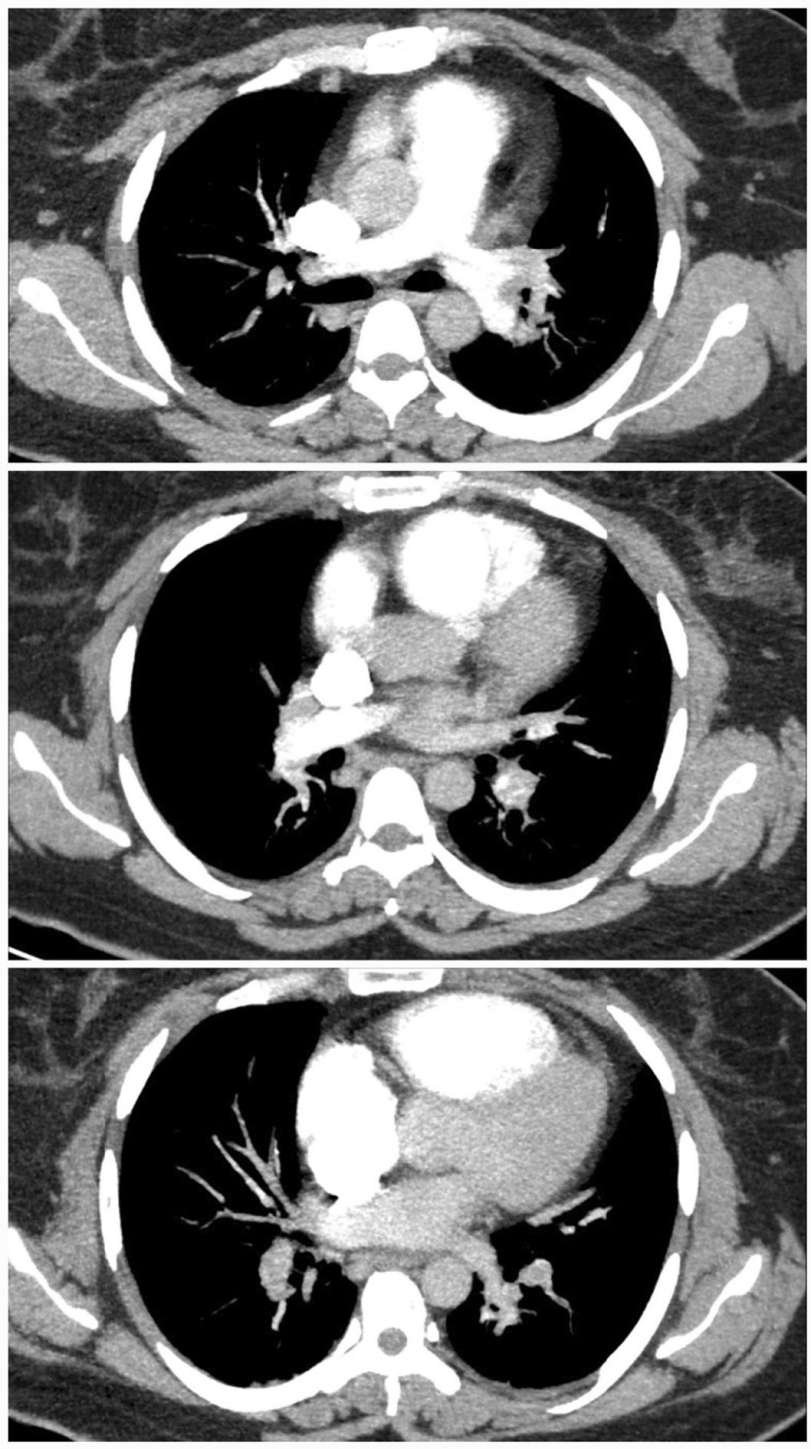
Multiple filling defects in bifurcation of the main bilateral pulmonary arteries which extends to segmental and lobar branches in favor of massive PE.

Spiral CT angiography of pulmonary vessels with contrast (PE protocol) showed multiple filling defects in the bifurcation of main bilateral pulmonary arteries which extended to segmental and lobar branches of both sides (confirmatory of massive PE). The findings of spiral CT angiography are shown in Figure 1.

In consultation wtih the cardiac surgery team, we decided that surgical thrombectomy would not benefit the patient as the thromboses were placed in the segmental vasculature. Therefore, the patient was transferred to the intensive care unit. After consulting with vascular surgery and cardiology teams, we started alteplase (100 mg continuous intravenous infusion over 2 h) with caution. The patient went under close observation, including abdominopelvic sonography (for early detection of intraabdominal leakage or bleeding), echocardiography, neurologic examinations, and laboratory follow‐ups. One hour after alteplase, her JP drain started to discharge blood (about 2 L in the first 24 h) along with bloody vaginal discharge, needing three bags of packed cells and three bags of fresh frozen plasma to be transfused. Although she initially was actively bleeding, it significantly decreased over the next 4 days. On postoperative day (POD) 4, she became hemodynamically stable with a normal abdominal exam and tolerated the diet. Thereafter, we started therapeutic heparin (800 IU/h continuous intravenous infusion) for 3 days. On POD 7, the bleeding stopped, and she was transferred to the surgery ward, where we switched from heparin to rivaroxaban as per the cardiologists' recommendation (15 mg twice daily). Then, she was observed for a single day and discharged from the hospital.

The medical team followed her after discharge until the submission of this paper (12 months). She experienced an uneventful postoperative period while losing 40 kg of her weight. On follow‐ups, she developed iron deficiency anemia, which is being treated with intravenous iron supplements. Hematologic evaluations for thrombotic tendencies, including factor V Leiden, protein C, and protein S were all unremarkable.

## 3 DISCUSSION

Candidates for bariatric surgery naturally amass multiple risks of thrombotic events. They suffer from obesity, chronic venous insufficiency, a recent surgery, and usually are less physically active.^6^ On the other hand, although these thromboembolic events are not common, they are highly detrimental and mostly present in the first 30 PODs.^2^, ^7^, ^8^ Nevertheless, unfortunately, there is not yet an established global consensus on thromboprophylaxis in these patients. The literature lacks an optimum drug, dosage, and duration for pharmacologic thromboprophylaxis due to a lack of class I evidence.

The presentation of PE is unspecific, which makes it difficult to diagnose. In this condition, the most important differential diagnoses in patients undergoing bariatric surgery are postoperative bleeding, anastomotic leakage, and myocardial infarction. Differentiation between these diagnoses is highly time‐sensitive. In our case, we decided to include a spiral abdominopelvic CT scan, spiral CT angiography, abdominopelvic ultrasonography, and echocardiography. We believed these evaluations would help narrow the differential diagnosis as quickly as possible. Then, we followed the therapeutic effects and potential adverse events through daily follow‐ups with abdominopelvic ultrasonography and echocardiography.

Massive PE is a serious complication and requires rigorous treatment. Careful clinical assessments must include proper risk stratification since it will influence both diagnostic and therapeutic decision‐making. In this regard, Aminian et al. have proposed a 30‐day post‐bariatric surgery DVT risk factor stratification model using data from 91,963 individuals.^9^ They found that almost 20% of post‐surgery DVT events occurred before patients were discharged. According to their findings, congestive heart failure, paraplegia, dyspnea at rest, and reoperation were associated with the highest risk of 30‐day DVT. Although it is plausible to hypothesize that certain DVT risk factors observed in patients who have undergone bariatric surgery may also be linked to heightened DVT risk in those undergoing endoscopic bariatric therapies, the absence of relevant data impedes definitive conclusions.^10^ Administration of systemic thrombolytics has been shown to resolve symptoms rapidly.^11^ However, systemic thrombolytics are controversial in bariatric surgery patients because they can adversely cause life‐threatening complications such as anastomotic leakage and intraabdominal bleeding; therefore, it is considered relatively contraindicated.^6^ Considering the debate mentioned earlier, massive PE appears to be a significant challenge to manage in bariatric surgery postoperative settings. However, considering the potentially fatal outcome of massive PE, we decided to take the risk of systemic thrombolytic, which was beneficial to the patient.

## 4 CONCLUSION

PE is uncommon in patients who undergo bariatric surgery, but if left untreated, it increases mortality risk and long‐term morbidity. On the other hand, systemic thrombolytic administration as a standard therapeutic approach is considered relatively contraindicated in massive PE that occurs shortly after surgery. Until the development of sufficient evidence, it is reasonable to approach thromboembolic events based on the individual and medical team's discretion.

## AUTHOR CONTRIBUTIONS

**Nader Moeinvaziri:** Conceptualization; writing – review and editing. **Alireza Sadeghi:** Conceptualization; visualization; writing – original draft; writing – review and editing.

## FUNDING INFORMATION

None.

## CONFLICT OF INTEREST STATEMENT

The authors have no conflict of interest to declare.

## CONSENT

A written consent was received from the patient and her family.

## Data Availability

The data used in this research will be provided by the corresponding author by reasonable request.
